# Dormant but Active: Chilling Accumulation Modulates the Epigenome and Transcriptome of *Prunus avium* During Bud Dormancy

**DOI:** 10.3389/fpls.2020.01115

**Published:** 2020-07-17

**Authors:** Karin Rothkegel, Paula Sandoval, Esteban Soto, Lissette Ulloa, Anibal Riveros, Victoria Lillo-Carmona, Javier Cáceres-Molina, Andrea Miyasaka Almeida, Claudio Meneses

**Affiliations:** ^1^ Centro de Biotecnología Vegetal, Facultad Ciencias de la Vida, Universidad Andrés Bello, Santiago, Chile; ^2^ Centro de Genómica y Bioinformática, Facultad de Ciencias, Universidad Mayor, Santiago, Chile; ^3^ FONDAP, Center for Genome Regulation, Universidad Andrés Bello, Santiago, Chile

**Keywords:** sweet cherry, Rosaceae, DNA methylation, chilling requirement, cold acclimation

## Abstract

Temperate deciduous fruit tree species like sweet cherry (*Prunus avium*) require long periods of low temperatures to trigger dormancy release and flowering. In addition to sequence-based genetic diversity, epigenetic variation may contribute to different chilling requirements among varieties. For the low chill variety ‘Royal Dawn’ and high chill variety ‘Kordia’, we studied the methylome of floral buds during chilling accumulation using MethylC-seq to identify differentially methylated regions (DMRs) during chilling hours (CH) accumulation, followed by transcriptome analysis to correlate changes in gene expression with DNA methylation. We found that during chilling accumulation, DNA methylation increased from 173 CH in ‘Royal Dawn’ and 443 CH in ‘Kordia’ and was mostly associated with the CHH context. In addition, transcriptional changes were observed from 443 CH in ‘Kordia’ with 1,210 differentially expressed genes, increasing to 4,292 genes at 1,295 CH. While ‘Royal Dawn’ showed approximately 5,000 genes differentially expressed at 348 CH and 516 CH, showing a reprogramming that was specific for each genotype. From conserved upregulated genes that overlapped with hypomethylated regions and downregulated genes that overlapped with hypermethylated regions in both varieties, we identified genes related to cold-sensing, cold-signaling, oxidation-reduction process, metabolism of phenylpropanoids and lipids, and a MADS-box *SVP-like* gene. As a complementary analysis, we used conserved and non-conserved DEGs that presented a negative correlation between DNA methylations and mRNA levels across all chilling conditions, obtaining Gene Ontology (GO) categories related to abiotic stress, metabolism, and oxidative stress. Altogether, this data indicates that changes in DNA methylation precedes transcript changes and may occur as an early response to low temperatures to increase the cold tolerance in the endodormancy period, contributing with the first methylome information about the effect of environmental cues over two different genotypes of sweet cherry.

## Introduction

During winter, perennial fruit trees from temperate regions face unfavorable environmental conditions like low temperatures. As a response mechanism, the tree generates protective structures called buds, which contain the meristematic tissue responsible for initiating the development of flowers and leaves (Fadón et al., 2015). Later in autumn, the tree ceases its visible growth and enters into dormancy, an adaptative process that sense the environmental cues to increase cold tolerance and to avoid flowering in winter (Lloret et al., 2018). According to Lang (1987), dormancy can be classified according to the physiological state into paradormancy, endodormancy and ecodormancy. Paradormancy refers to growth inhibition due to apical dominance, while endodormancy corresponds to an endogenous inhibition from the meristem; and growth inhibition due to unfavorable temperatures is referred to as ecodormancy (Lang, 1987).

Sweet cherry (*Prunus avium* L.) belongs to the Rosaceae family and is cultivated in areas of temperate climate, entering into dormancy in autumn to survive the low temperatures of winter. During endodormancy, in *P. avium* and other Rosaceae species, the prolonged exposition to low temperatures in winter and the fulfillment of a chilling requirement (CR), is critical to ensure an optimal flowering in spring and is considered to be specific for each variety or genotype (Campoy et al., 2011). However, with warmer winters due to climatic change, the CR of high chill varieties may not be fulfilled, leading to a delay in flowering and therefore, productivity problems (Campoy et al., 2011). Because of this, in some areas it is necessary the use of chemicals to improve the break of dormancy (Erez et al., 2008). On the other side, low chill varieties have the risk of completing this CR earlier in winter, being exposed to spring frost.

In order to adapt fruit crops to the constantly changing environment, it is necessary to understand the molecular basis of dormancy. Previous studies of dormancy have been focused in the genetic control, showing that CR is a major determinant for flowering date in peach (*Prunus persica*), sweet cherry, and almond (*Prunus dulcis*) (Fan et al., 2010; Sánchez-Pérez et al., 2011; Castede et al., 2014). In peach, quantitative trait loci (QTL) have shown that bloom date is highly variant across years because of the interaction between genotype and environment, being the chilling and heat accumulation the major sources of environmental effects (Fan et al., 2010). The authors suggested that the variable temperatures interact with different genotypes, affecting their CR and therefore, blooming.

At the transcriptomic level, studies in *Populus*, leafy spurge (*Euphorbia esula* L.), Japanese pear (*Pyrus pyrifolia* Nakai) and peach, showed changes in processes involved with cold acclimation, responses to phytohormones, cellular transport, carbohydrate metabolism, response to oxidative stress, DNA methylation and histone modifications (Horvath et al., 2008; Jiménez et al., 2010; Bai et al., 2013; Howe et al., 2015). A recent study in sweet cherry has determined that buds in the stages of organogenesis, paradormancy, endodormancy and ecodormancy, can be defined by their expression profile. In this sense, before dormancy, an increase in the expression of *DORMANCY ASSOCIATED MADS-box* genes (*DAM*), floral identity genes and developmental genes was observed. Later in endodormancy, the authors observed an overrepresentation of genes that participate in the cold-response, abscisic acid (ABA) and oxidation-reduction processes (Vimont et al., 2019).

Epigenetic mechanisms involving histone modifications, DNA methylation and small non-coding RNAs are suggested as regulators of dormancy in a similar way as vernalization in *Arabidopsis* (Amasino, 2004). In peach, histone modifications observed in *DAM6*, were associated with gene repression after dormancy release (Leida et al., 2012). In addition, peach miRNAs were found to be differentially expressed between dormant and non-dormant leaf buds, some of them colocalizing with QTLs for CR (Barakat et al., 2012). In chestnut (*Castanea sativa*), global levels of DNA methylation increased in dormant buds in comparison to non-dormant buds, while in almond, the identification of differential methylation states in response to chilling accumulation provided information about methylation markers for flowering (Santamaría et al., 2009; Prudencio et al., 2018). In plants, DNA methylation occur in three different contexts: CpG, CHG and CHH, were H can be either cytosine, thymine or adenine (Law and Jacobsen, 2010). In sweet cherry, an increase in DNA methylation in all cytosine contexts was associated with an increase in the abundance of matching siRNAs in the promoter of a *MADS-box* gene (*MADS1*), homologous to the peach *DAM* genes, at the fulfillment of CR (Rothkegel et al., 2017).

Despite this, a better understanding of the molecular control of dormancy still needs to be established. In this study, the main objective is to elucidate the global changes in DNA methylation and transcript levels during chilling accumulation in dormant buds of sweet cherry varieties contrasting for CR. For this, we used whole-genome bisulfite sequencing (MethylC-seq), followed by the additional sequencing of vectors and amplicons that comprise differentially methylated regions as validation of the methylation pattern at specific loci. We used RNA-seq to analyze transcriptomic profiles modulated by chilling accumulation and integrated MethylC-seq and RNA-seq for the identification of biological processes and molecular pathways that may participate in dormancy regulation. Finally, our work contributes with the first epigenomic data at the DNA methylation level for sweet cherry, also providing additional information about the interaction between environment and genotype in the Rosaceae family.

## Materials and Methods

### Plant Material

**Figure 1:**
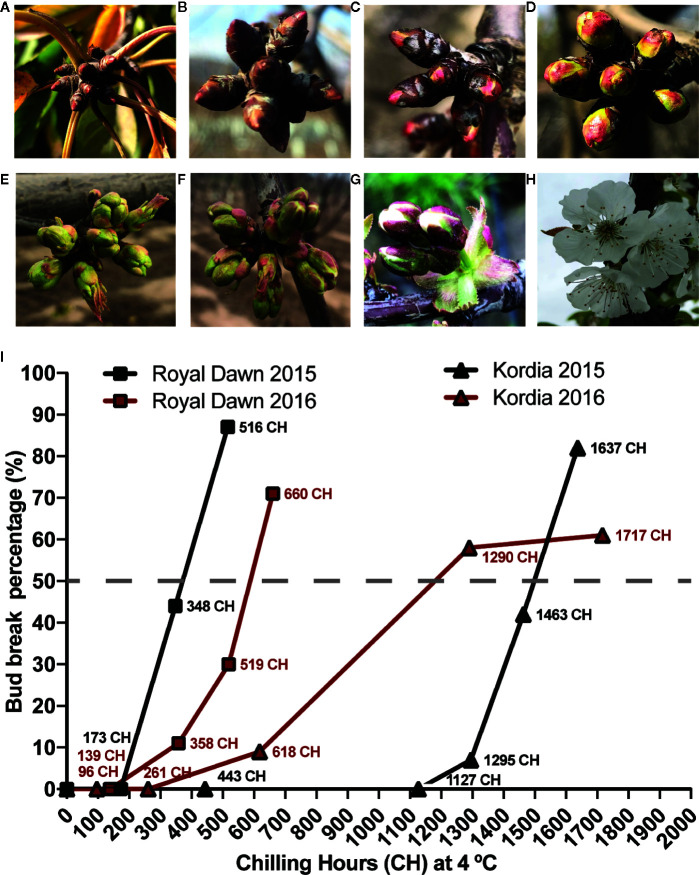
Developmental growth stages of flower buds and sampling conditions during chilling accumulation in sweet cherry varieties according to the BBCH scale (*Biologische Bundesantalt, Bundessortenamt und Chemische Industrie*) (Fadón et al., 2015). **(A)** Paradormant buds and senescent leaves in autumn; **(B)** Endodormant buds during chilling accumulation in winter; **(C)** Inflorescence buds swelling and breaking from ecodormancy (BBCH 51 stage); **(D)** bud burst in spring; **(E)** Inflorescence enclosed by green scales; **(F)** Three to four inflorescences generated from a single flower bud; **(G)** Flower pedicel elongation; **(H)** Flowering; **(I)** Two seasons of bud break percentage during CH accumulation and sampling points for DNA methylation and transcriptome analysis in the low chill variety ‘Royal Dawn’ and high chill variety ‘Kordia’. The dashed line represent a complete chilling requirement, indicated as a 50% or more of bud break.

*P. avium* L. var. ‘Royal Dawn’ was cultivated and sampled during 2015 and 2016 from the commercial orchard ‘Agrícola Garcés’ located at San Francisco de Mostazal, Región de O’Higgins, Chile (33° 59’ 53” S; 70° 41’ 38” W). Adult trees of ‘Kordia’, a variety that needs more chilling hours (CH) accumulation to flower, were cultivated in two different fields. During 2015, we sampled trees cultivated in Pontificia Universidad Católica de Valparaíso, Quillota (32° 53’43.6” S; 71° 12’ 34”W), and during 2016 we sampled trees from ‘Agrícola Garcés: Fundo Entre Ríos’, O’Higgins region, Chile (34° 41’ 10.4” S; 70° 52’ 23.5” W). Cuttings containing around four to six clusters of floral buds were randomly collected before chilling accumulation (0 CH) and stored at −80°C. In winter, approximately thirty cuttings, considering ˜30 trees per variety, were sampled and stored in a cold chamber at 4°C without light for chilling accumulation. Every seven days, six cuttings were collected from the cold chamber. Three of these cuttings were rehydrated by re-cutting their basal end under water and placed in a greenhouse (25°C and 16/8 h day/night) for the estimation of bud break in the BBCH (*Biologische Bundesantalt, Bundessortenamt und Chemische Industrie*) 51 stage (**Figure 1**). In parallel, from the remaining three cuttings, four to six floral buds from each cutting were considered as three biological replicates for each sample point (**Figure 1I**). Buds were stored at −80°C for later use in MethylC-seq and RNA-seq.

### Measurement of Chilling Requirement (CR)

The phenological stages of dormancy and flowering of sweet cherry varieties ‘Royal Dawn’ and ‘Kordia’ were analyzed according to the BBCH scale (**Figure 1**) (Fadón et al., 2015). The CR necessary for bud break was measured for each variety as chilling hours (CH; number of hours at a temperature below 7.2°C) (Weinberger, 1950). Every seven days, ‘Royal Dawn’ and ‘Kordia’ cuttings sampled from the cold chamber were placed in water under favorable conditions (25°C and 16/8 h day/night) in a greenhouse. After 14 days in the greenhouse, the phenological state of the floral buds was analyzed and the CR was considered to be completed when at least 50% of the buds were swelling and began to show sepals in BBCH 51 stage (**Figure 1C**).

### MethylC-Seq

Bisulfite treatment was carried out for season 2015 from genomic DNA of floral buds with different CH accumulation from ‘Royal Dawn’ (0 CH, 173 CH, 348 CH and 516 CH) and ‘Kordia’ (0 CH, 443 CH, 1,295 CH and 1,637 CH). DNA was extracted using the DNeasy Plant mini kit (QIAGEN, Germantown, MD, USA) according to manufacturer’s instructions. DNA integrity was assessed in a 1.5% (p/v) agarose gel and concentration was determined by Qubit Fluorometer (Thermo Fisher Scientific, Waltham, MA, USA). A hundred nanograms of sample DNA was used for bisulfite treatment with the EZ DNA methylation gold kit (Zymo, Irvine, CA, USA) as previously described (Rothkegel et al., 2017). Twenty-four indexed and strand specific libraries were generated considering: two varieties, four sampling points and pooled buds from three cuttings as biological replicates. Ten nanograms of untreated DNA from ‘Royal Dawn’ and ‘Kordia’ were used as negative control libraries. All libraries were obtained with the TruSeq DNA Methylation kit (Illumina, San Diego, CA, USA) according to manufacturer’s instructions. Validated libraries by Qubit Fluorometer and Fragment Analyzer (Advanced Analytical Technologies, Ankeny, IA, USA) were sequenced in HiSeq 2500, 2 × 125 bp Paired-end mode (Illumina, San Diego, CA, USA). Raw data is available at NCBI sequence read archive (PRJNA610988 and PRJNA610989).

### Processing and Alignment of Bisulfite Reads

The partial genome of ‘Royal Dawn’ and ‘Kordia’ was generated with Bowtie 2 mapping the non-bisulfite treated libraries against the available data of *P. avium* as reference (Shirasawa et al., 2017) (**Table S2**). Adapters, low quality reads and clonal reads from each library were filtered with Trim Galore (https://www.bioinformatics.babraham.ac.uk/projects/trim_galore/). Filtered reads of bisulfite treated libraries were mapped to the partial genome of each variety using Bismark (Krueger and Andrews, 2011), with no mismatches. The methylation state in CpG, CHG and CHH was determined from the aligned reads using Bismark and MethylPipe (Kishore et al., 2015), and exported to Seqmonk software (Krueger and Andrews, 2011).

### Methylation Level of Control Genes

To study the predictions obtained with MethylC-seq in a non-model species, we searched for a gene that should be transcriptionally active and a transposable element that should be highly methylated. Genomic DNA from floral buds of season 2015 was bisulfite treated using the EZ DNA Methylation gold kit as mentioned above, followed by amplification with ZymoTaq Polymerase (Zymo, Irvine, CA, USA) using primers for a putative *ACTIN-BINDING COMPONENT* (Pav_sc0002118.1_g070.1.mk) and a transposable element (Pav_ sc0000224.1_g040.1.br) (**Table S1**). The PCR product was cloned into a pGEM^®^-T vector (Promega, Madison, WI, USA), and ten clones per condition were sequenced by the Sanger method in Macrogen, Seoul, Korea. The obtained sequences were analyzed through Kismeth (http://katahdin.mssm.edu/kismeth/revpage.cl).

### Identification of Differentially Methylated Regions

From cytosines covered from at least five reads, a sliding-window approach of 100 bp was used to analyze regions in the partial sweet cherry genome. The methylation state was calculated as log2 enrichment (log2 ratio of the observed base density in the region divided by the overall base density in the sample), and windows with less than 20 methylated cytosines were discarded. An ANOVA analysis (p-value <0.01) was used to obtain significant differences between windows from the four chilling conditions (0 CH, 173 CH, 348 CH and 516 CH for ‘Royal Dawn’; 0 CH, 443 CH, 1,295 CH and 1,637 CH for ‘Kordia’). P-value was later adjusted using a Benjamini and Hochberg correction (FDR <0.01). In addition, windows with differences of at least log2 fold-change >3 in their methylation state between two of the four conditions were identified as differentially methylated. A DMR that overlaps with a gene, including 2,000 bp upstream and downstream, was annotated as a differentially methylated gene. Subclusters of methylation profiles from DMRs were obtained considering the log2 enrichment and Kmer with MeV software.

### Amplicon Bisulfite Sequencing (ABS) of Targeted Regions

Because of the lack of a reference genome, we complemented MethylC-seq with ABS using sampling points from 2015 and 2016 (**Figure 1I**). From DNA that was previously treated with bisulfite, a first PCR was performed with primers specific to each methylated region (**Table S1**) using ZymoTaq Polymerase. Twenty microliters of magnetic beads AMPure XP (Beckam-Coulter, Oakley Court, UK), were added to 25 μl of PCR product and incubated 15 min at room temperature in a magnetic stand (Thermo Fisher Scientific, Waltham, MA, USA). Supernatant was discarded and with the plate in the magnetic stand, two washes of 200 μl of freshly made ethanol 80% were added and incubated for 30 s. Ethanol was discarded and the plate was set to dry for 15 min at room temperature. The stand was removed from the magnetic plate, the pellet was resuspended in 22.5 μl of resuspension buffer (TE buffer, Tris-HCl pH 8.5) and incubated for 2 min. The stand was located in the magnetic plate and incubated for 1 min or until liquid was clear. Twenty microliters were transferred to a new tube and 2 μl were used as template for a second PCR with 10 μl of GoTaq^®^ Green Master Mix (Promega, Madison, WI, USA), 10 μM of Nextera indexed adapters (Illumina, San Diego, CA, USA) and nuclease free water to a final volume of 20 μl. The PCR program considers an initial denaturation at 94°C for 1 min, followed by eight cycles at 95°C for 30 s, annealing of 68°C for 30 s, extension at 72°C for 30 s and a final extension of 5 min. Nuclease free water was added to the PCR product to reach a final volume of 50 μl. Fifty-six microliters of AMPure XP beads were added to purify the PCR product as described above but using 27.5 μl of resuspension buffer in the final step. The purified libraries were validated with Qubit Fluorometer using a High-Sensitivity DNA kit (Thermo Fisher Scientific) and Fragment Analyzer. Validated libraries were sequenced with MiSeq in 2 × 250 bp Paired-end mode (Illumina). Filtered reads were mapped to their reference with Bismark as previously described.

### Total RNA Sequencing

Total RNA was extracted from floral buds of season 2015 considering three replicates of 0, 348, and 516 CH for ‘Royal Dawn’; and 0, 443, 1,295, and 1,637 CH for ‘Kordia’. RNA was extracted with PureLink™ Plant RNA Reagent (Thermo Fisher Scientific) according to manufacturer’s instructions. Quality of RNA was assessed by capillary electrophoresis and Qubit RNA BR Assay kit (Thermo Fisher Scientific). One microgram of RNA was used for construction of strand-specific libraries with the TruSeq Stranded mRNA kit (Illumina), and validated libraries were sequenced in HiSeq 4000, 2 × 100 bp Paired-end mode (Macrogen, Seoul, Korea). Raw data is available at NCBI sequence read archive (PRJNA611731 and PRJNA611733).

### Data Analysis of RNA-Seq

Paired-end reads (100 bp) were trimmed with Trim Galore and mapped to the partial genome of *P. avium* (Shirasawa et al., 2017), using Spliced Transcripts Alignment to a Reference (STAR; Dobin et al., 2013). Filtered reads were normalized as trimmed mean of M-values (TMM) and used for differentially expressed gene (DEG) analysis with EdgeR considering a False Discovery Rate (FDR) <0.01 and a two-fold-change (Robinson et al., 2010). Subclusters of co-expressed genes (normalized with FPKM) were obtained with Kmer and MeV software.

### Integration of MethylC-Seq and RNA-Seq Data

From previously obtained DMRs and DEGs, Venn diagrams were generated to obtain an overlap between the loci of methylations and transcripts. From hypermethylated regions associated with downregulated genes and hypomethylated regions with upregulated genes, we selected only the conserved patterns between varieties and represented them as a heatmap. As an additional and complementary analysis, we used conserved and non-conserved DMRs close to DEGs (upregulated and downregulated genes) for up to 2,000 bp upstream and downstream to determine the correlation value between DMR and transcript levels across all CH conditions. Those genes that presented a negative correlation value of −0.5 or less between the methylation and transcript levels were used for Gene Ontology (GO) analysis (FDR <0.01) with BiNGO from Cytoscape version 3.0.3 (http://apps.cytoscape.org/apps/bingo).

### Real Time qPCR Analysis

One microgram of total RNA was treated with DNase I (Thermo Fisher Scientific), followed by cDNA synthesis with SuperScript™ first-strand synthesis system and oligo dT primers (Thermo Fisher Scientific), according to the standard protocol. Each cDNA sample was diluted 1:10 with nuclease free water before use. Master mix for RT-qPCR consisted of KAPA SYBR^®^ FAST qPCR master mix (Kapa Biosystems, Wilmington, MA, USA), 10 μM of forward primer (**Table S1**), 10 μM of reverse primer (**Table S1**), ROX dye, template cDNA and PCR-grade water for a final volume of 10 μl. The RT-qPCR assay was performed in an AriaMx real-time PCR system (Agilent Technologies, Santa Clara, CA, USA). All RT-qPCR assays were performed using three biological and three technical replicates. Expression profiles were normalized to *Pavβ-ACTIN* gene and relative expression was obtained based in the ΔCT method.

## Results

### Dormancy and Chilling Requirement for Contrasting Varieties of Sweet Cherry (*P. avium*)

In order to estimate the chilling requirement of ‘Royal Dawn’ and ‘Kordia’ during season 2015 and 2016, we sampled cuttings with floral buds in a paradormant state before cold accumulation (0 CH) (**Figure 1A**), and endodormant buds with different CH accumulation (**Figure 1I**). Bud break was determined when 50% or more of flower buds were in BBCH 51 stage (**Figure 1C**), which is considered to be the minimum chilling requirement (CR) for normal flowering. Considering this, for ‘Royal Dawn’ trees, we estimated that CR was completed at 516 CH (2015) and 660 CH (2016) (**Figure 1I**). ‘Kordia’ trees needed a higher chilling accumulation to complete the CR, observed at 1,637 CH (season 2015) and 1,290 CH (season 2016) (**Figure 1I**).

### Genome Wide Sequencing of DNA Methylations in Contrasting Varieties for CR During Chilling Accumulation

**Figure 2:**
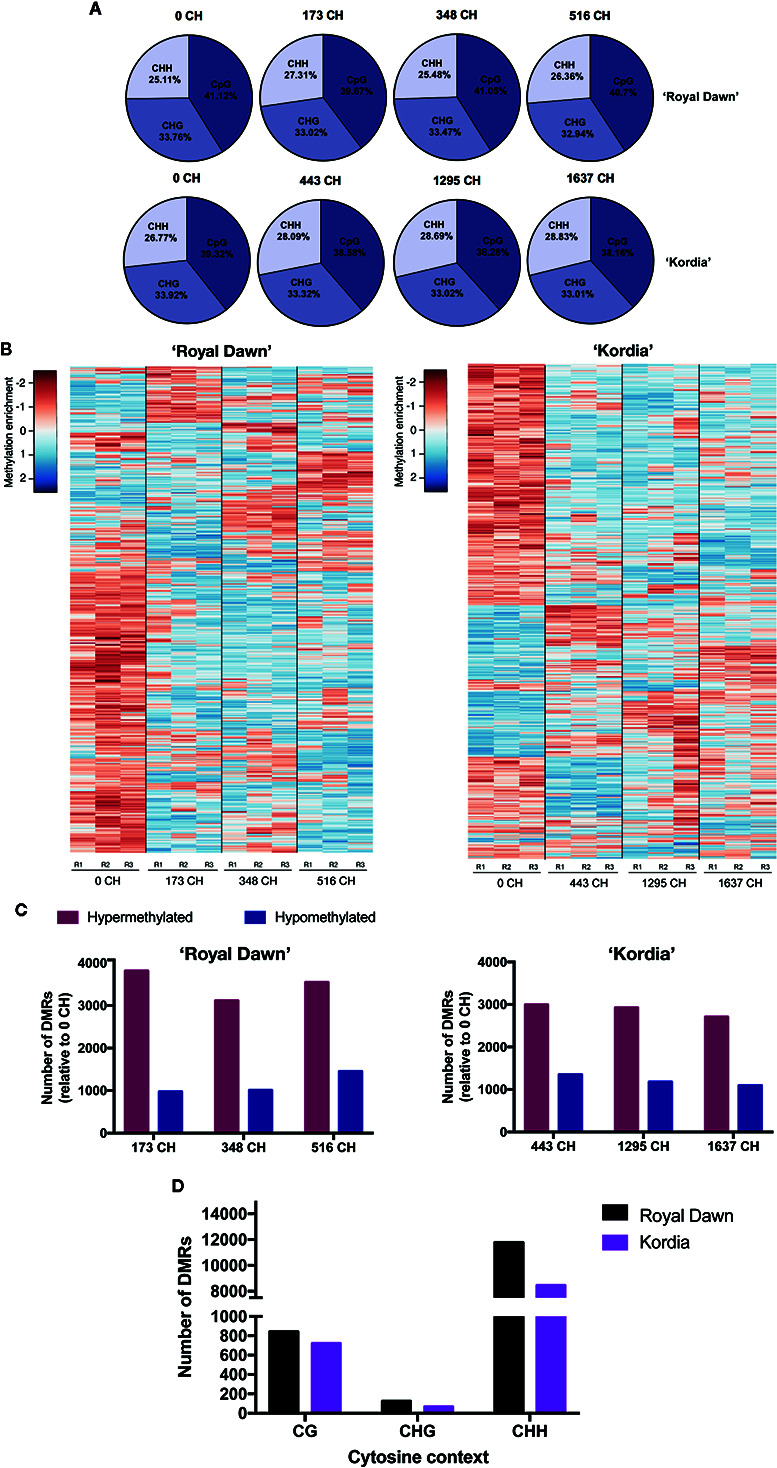
Methylation changes across chilling accumulation in floral buds of ‘Royal Dawn’ and ‘Kordia’. **(A)** Relative levels of methylated cytosines in the contexts CG, CHG and CHH. **(B)** Heatmap of DNA methylation levels for 1,000 DMRs (log2FC >3; FDR <0.01; all cytosine contexts) identified from comparisons of 100-bp windows among 0, 173, 348 and 516 CH in ‘Royal Dawn’; and comparisons among 0, 443, 1,295 and 1,637 CH in ‘Kordia’. **(C)** Number of hypermethylated and hypomethylated regions from 173 vs 0 CH (hyper = 3,814; hypo = 975), 348 vs 0 CH (hyper = 3,115; hypo = 1,010), and 516 vs 0 CH (hyper = 3,546; hypo =1,451) in ‘Royal Dawn’; and number of regions at 443 vs 0 CH (hyper = 2,990; hypo = 1,352), 1,295 vs 0 CH (hyper = 2,919; hypo = 1,179) and 1,637 vs 0 CH (hyper = 2,703; hypo = 1,094) in ‘Kordia’. **(D)** Number of DMRs in each cytosine context from comparisons between 173, 348 and 516 CH against 0 CH in ‘Royal Dawn’ (CG, n = 843; CHG, n = 126; CHH, n = 11,786); and comparisons between 443, 1,295 and 1,637 CH against 0 CH in ‘Kordia’ (CG, n = 723; CHG, n = 67; CHH, n = 8,448). H = C, T or A.

For MethylC-seq of varieties ‘Royal Dawn’ and ‘Kordia’, we isolated genomic DNA from floral buds exposed to different CH accumulation of season 2015 (**Figure 1I**). From uniquely mapped reads (**Table S2**), we obtained the relative and absolute levels of methylated cytosines for the different contexts (CG, CHG and CHH; H = C, T or A) (**Figure 2A**, **Table S3**). The relative levels of methylated cytosines showed that in ‘Royal Dawn’, a 40–41% belong to the CpG context, while a 33% corresponds to CHG and a 25–27% to the CHH context (**Figure 2A**). The same tendence was observed in ‘Kordia’, where 38–39% corresponds to CpG, 33% to CHG and 27–29% to CHH (**Figure 2A**). To study the epigenetic variation during chilling accumulation in dormancy, we used the methylation calls and quantified them as log2enrichment to perform a sliding-window approach of 100 bp (FDR <0.01) and search for differentially methylated regions (DMRs) across the four chilling conditions of each variety. Considering all cytosine contexts, we identified 9,600 DMRs in ‘Royal Dawn’ (**Table S4**) and 8,535 in ‘Kordia’ (**Table S5**). For both varieties, we grouped these DMRs in 16 subclusters according to the average methylation level and pattern (**Tables S6 and S7**). Considering only the top 1,000 DMRs that showed a highest variance value in their methylation enrichment among the four chilling conditions, changes in the methylation level were mainly between 0 CH and 173 CH for ‘Royal Dawn’ and between 0 CH and 443 CH for ‘Kordia’ (**Figure 2B**). In addition to this, a higher number of the overall DMRs was associated to hypermethylation in both varieties, however ‘Royal Dawn’ showed 3,115–3,814 of hypermethylated regions compared to ‘Kordia’ with 2,703–2,990 (**Figure 2C**). To identify the most variable cytosine context to be methylated, we searched for DMRs considering only CpG, CHG or CHH (**Figure 2D**). The highest variation in methylation was observed in the CHH context, with 11,786 (CHH), 126 (CHG) and 843 (CpG) DMRs in ‘Royal Dawn’; the same tendency was observed for ‘Kordia’ with 723 (CpG), 67 (CHG) and 8,448 (CHH) DMRs (**Figure 2D**).

To study the results obtained with MethylC-seq, we searched for a gene that should be transcriptionally active (*e.g.* related to a housekeeping gene), and on the other hand a transposable element that should be highly methylated (**Supplementary Figure 1**). From the genomic data, we synthetized primers and with the same samples used previously, we performed bisulfite treatment followed by cloning and sequencing of 10 clones per condition (**Supplementary Figures 1 and 2**). The sequencing of clones from ‘Kordia’ and ‘Royal Dawn’ revealed that a putative *ACTIN-BINDING COMPONENT* (Pav_sc0002118.1_g070.1.mk) possess gene body methylation in the CpG context for all CH accumulations, while the transposable element (Pav_ sc0000224.1_g040.1.br) is highly methylated in the three cytosine context and maintained during chilling accumulation.

In addition, to validate targeted DMRs with higher depth, we implemented the sequencing of small size amplicons (size <350 bp) containing a DMR flanked by sequencing adapters. From the MethylC-seq data we analyzed a DMR of 180 bp that is located ˜1,600 bp downstream a gene annotated as a *2-ALKENAL REDUCTASE NADP*(+)-*DEPENDENT* (**Supplementary Figure 3**). From MethylC-seq data, in ‘Royal Dawn’ we observed an increase in the methylation level regarding to 0 CH, starting from 0.7 log2enrichment at 175 CH, increasing to 2.7 at 516 CH. In ‘Kordia’ there was a decrease of the levels of methylation at 443 CH, increasing later at 1,637 CH (**Supplementary Figure 3A**). Concomitant with this, the amplicon bisulfite sequencing (ABS) of the targeted region in ‘Royal Dawn’ showed an increase of the methylation level in the CHH context (from 76 to 82%), reaching ‘Kordia’ levels during chilling accumulation for two consecutive seasons (2015 and 2016). In ‘Kordia’, ABS showed a decrease from 87 to 84% at 1565 CH for the CpG context and a decrease from 84 to 79% at 557 CH, followed by an increase of 81% at 1565 CH for the CHG context (**Supplementary Figure 3B**). On the other side, the transcript level for *2-ALKENAL REDUCTASE NADP*(*+*) started with a higher expression in ‘Kordia’ at 0 CH, followed by a decrease in both varieties until a complete CR (**Supplementary Figure 3C**).

### Sequencing the Transcriptome of Contrasting *P. avium* Varieties for CR During Chilling Accumulation

**Figure 3:**
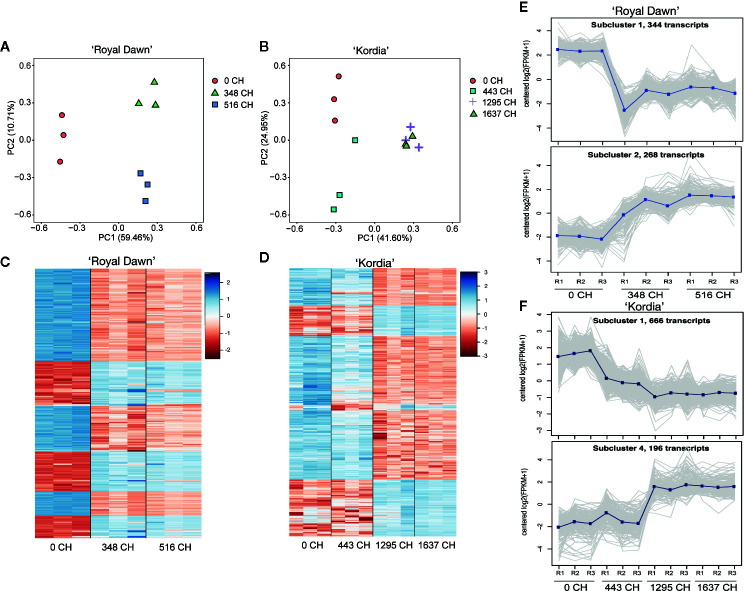
Transcriptome profile during chilling accumulation in ‘Royal Dawn’ and ‘Kordia’. **(A)** Principal component analysis of global gene expression with three biological replicates at 0, 348 and 516 CH in ‘Royal Dawn’; and **(B)** at 0, 443, 1,295 and 1,637 CH for ‘Kordia’. **(C)** Heatmap representing the 1,000 transcripts with most variance among the conditions in ‘Royal Dawn’ and **(D)** ‘Kordia’. Color key indicates the median of log2 TMM-normalized values and red to blue represents low to high levels of transcript, respectively. **(E)** Representative subcluster plots (2 out of 10) of the overall genes that increase or decrease their expression during chilling accumulation in ‘Royal Dawn’ and **(F)** subcluster plots (2 out of 11) of ‘Kordia’.

In order to identify transcripts that could be regulated by DNA methylation and chilling accumulation, we sequenced the transcriptome (RNA-seq) of dormant buds using samples of season 2015 (**Figure 1I**). In this case, 70–90% of the reads mapped uniquely, while 5–23% of the reads did not align (**Table S8**). From the uniquely mapped reads, we performed a principal component analysis (PCA) to determine if the overall gene expression was modulated by chilling accumulation (**Figures 3A, B**). For ‘Royal Dawn’, a 70.15% of the variability in gene expression was explained by PCA, in which all treatments were well separated according to their cold accumulation (**Figure 3A**). On the other side, for the high chill variety ‘Kordia’, PCA explains a 66.5% of the variability and could separate 0 CH, 443 CH and 1,295 CH, but placing together 1,295 CH and 1,637 CH, indicating a regulation that is specific for each variety (**Figure 3B**). Together with this, the expression level (log2 TMM-normalized values) of the top 1,000 transcripts were ranked according to their TMM variance value among the four chilling conditions. For ‘Royal Dawn’, changes in the overall gene expression were observed from 348 CH (**Figure 3C**), while in ‘Kordia’, differences in overall gene expression were observed from 443 CH but increasing at 1,295 CH (**Figure 3D**), indicating that gene expression is regulated depending on the variety. In addition, we performed a co-expression analysis to group the overall genes into subclusters according to their averaged expression levels (**Supplementary Figure 4**), obtaining 10 subclusters in ‘Royal Dawn’ and 11 in ‘Kordia’. Two subclusters that represented an increase and a decrease of the transcript levels during cold accumulation are shown in **Figures 3E, F**.

**Figure 4:**
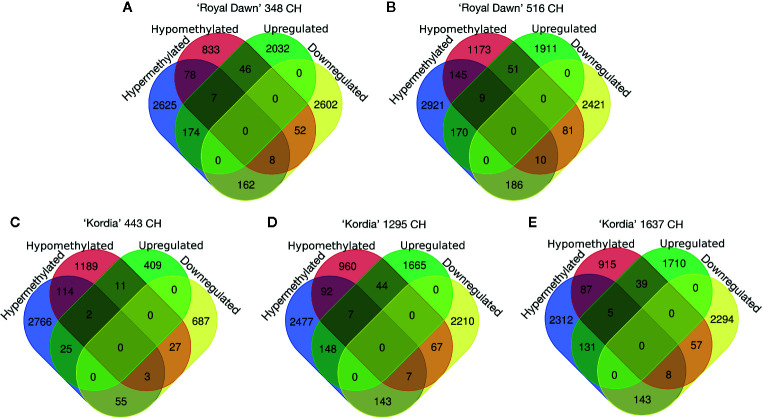
Venn diagrams representing the number of DEGs (upregulated and downregulated genes) that overlapped with DMRs (hypermethylations and hypomethylations). **(A)** Overlapping of DMRs and DEGs at 348 CH and **(B)** 516 CH for ‘Royal Dawn’. **(C)** Number of DMRs and DEGs that overlapped at 443 CH, **(D)** 1,295 CH and **(E)** 1,637 CH for ‘Kordia’.

For differentially expressed genes (DEGs), a two-fold difference (log2 ≥1) in the transcript counts and a false-discovery rate of 0.01 or less were used as threshold to compare the cold treatments vs 0 CH (no cold). With the DEGs generated from 348 vs 0 CH, 516 vs 0 CH (‘Royal Dawn’); and 443 vs 0 CH, 1,295 vs 0 CH, 1,637 vs 0 CH (‘Kordia’), we study the relationship between DNA methylation and gene expression during chilling accumulation. Initially, for ‘Royal Dawn’ we observed 5,083 (2,259 upregulated and 2,824 downregulated) genes that significantly change their expression at 348 CH, and 4,839 (2,141 upregulated and 2,698 downregulated) genes at 513 CH (**Figures 4A, B**). ‘Kordia’ started from 1,219 DEGs (447 up and 772 down), increasing to 4,291 DEGs (1,864 up and 2,427 down) at 1,295 CH and 4,387 DEGs (1,885 up and 2,502 down) at 1,637 CH, also coinciding with PCA results (**Figure 3B**).

To see if RNA-seq data was reflected in additional expression analysis, we made qPCRs in both varieties from the same RNA and studied *FLOWERING LOCUS T* as a gene associated to dormancy and flowering regulation. Genes that showed a DMR with increased methylation levels and correlated with transcript downregulation, *TRANSPARENT TESTA 1, FLAVONOL SYNTHASE*, *AMSH-like* and *β-GALACTOSIDASE*. Additionally, a gene that increased its expression with CH accumulation and was related to cold response, *COLD-SHOCK PROTEIN 2* (**Table S1**). All these genes showed similar patterns in RNA-seq and qPCR (**Supplementary Figure 5**).

### Integration of the Methylome and Transcriptome During Chilling Accumulation

Afterwards, when analyzing the overlap between DEGs and DMRs, we observed for ‘Royal Dawn’ that 174 and 162 DMRs were associated with hypermethylation that overlapped with genes that significantly increase and decrease their expression at 348 CH, respectively. At the same time, 46 and 52 hypomethylated regions overlapped with upregulated genes and downregulated genes, respectively (**Figure 4A**). At 516 CH, 170 and 186 hypermethylations coincided with upregulated and downregulated genes respectively, while 51 and 81 hypomethylated regions coincided with upregulated and downregulated genes (**Figure 4B**).

For ‘Kordia’, 25 and 55 hypermethylated regions were related to upregulated and downregulated DEGs at 443 CH, while 11 and 27 hypomethylations were associated to upregulated and downregulated genes, respectively (**Figure 4C**). With 1,295 CH, we observed an increase of 148 and 143 hypermethylations coincident with upregulated and downregulated genes, together with 44 and 67 hypomethylations associated to genes that increase and decrease their expression, respectively (**Figure 4D**). At 1,637 CH, 131 and 143 regions that increase their methylation level overlapped with genes that increase and decrease their expression, respectively. In addition, 39 and 57 regions that decreased their methylation levels overlapped with upregulated and downregulated genes, respectively (**Figure 4E**).

Regarding to DMRs that were associated to genes that do not change their expression in a significant manner (**Figure 4**), it can be observed that 2,703 hypermethylations and 911 hypomethylations were not related to DEGs in ‘Royal Dawn’ at 348 CH. This number increase with 3,066 hypermethylations and 1,318 hypomethylations at 516 CH. In ‘Kordia’, the opposite occurs were 2,880 hyper and 1,303 hypomethylated regions were observed at 443 CH, followed by a decrease of 2,569 hyper and 1,052 hypomethylations, coincident with the increase of DMRs overlapping with DEGs. Finally, at 1,637 CH, 2,399 hyper and 1,002 hypomethylated regions were observed (**Figure 4**).

### DMRs and DEGs Conserved in ‘Royal Dawn’ and ‘Kordia’

**Figure 5:**
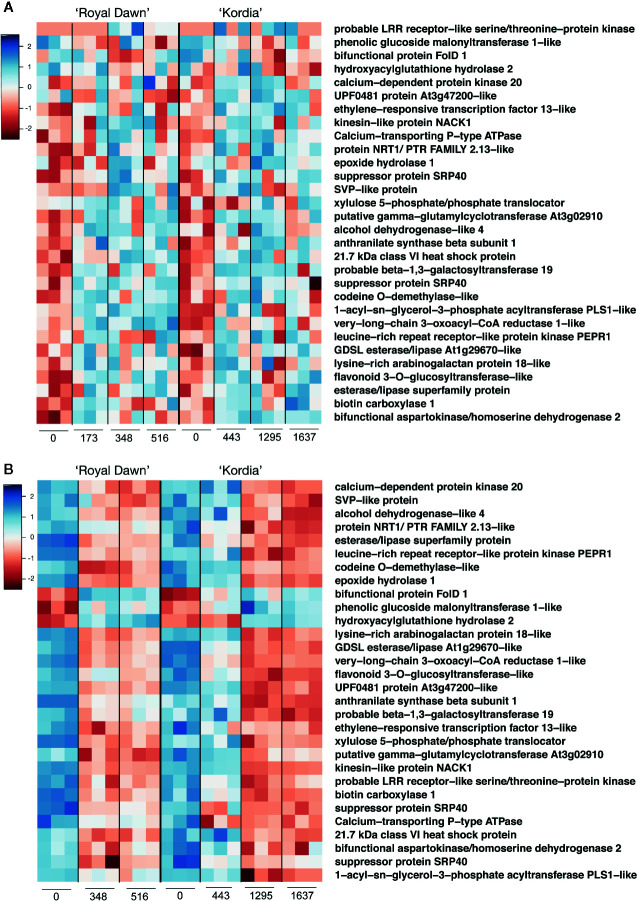
Representation of thirty genes that showed a conserved hyper/hypomethylation pattern that overlapped with down/upregulated genes. **(A)** Methylation levels (log2 enrichment) from DMRs related to hyper and hypomethylations at different CH accumulation points for ‘Royal Dawn’ and ‘Kordia’. **(B)** Transcript levels (log2 TMM-normalized values) from DEGs related to downregulated and upregulated genes during CH accumulation in ‘Royal Dawn’ and ‘Kordia’. Color key from red to blue represents low to high levels, respectively.

From the overlap between hypermethylated region/downregulated genes and hypomethylated region/upregulated genes (**Figure 4**), we searched for those patterns conserved in both varieties. From this analysis, we obtained thirty genes that showed a conserved pattern of methylations and expression in both varieties, indicating that most of these modifications are unique to each variety (**Figure 5**). The identified genes were related to a response to stress linked to the sensing and signal transduction of cold, such as two protein kinases, *ETHYLENE-RESPONSIVE TRANSCRIPTION FACTOR* *13*-like, one heat-shock protein and two proteins dependent of calcium (Ca^+2^). For example, *CALCIUM-TRANSPORTING P-type ATPase* showed an increase in the methylation levels from a DMR located downstream the gene, starting from 173 CH in ‘Royal Dawn’ and 443 CH in ‘Kordia’, coincident with the decrease in the transcript levels at 348 CH in ‘Royal Dawn’ and 443 CH in ‘Kordia’ (**Figure 5**).

Genes associated to an oxidation-reduction process, like 3 dehydrogenases and a dioxygenase *O-DEMETHYLASE*-like, were also identified. *FOLD1* is a mitochondrial dehydrogenase that in ‘Royal Dawn’ showed a decrease in methylations from a DMR located upstream the gene at 348 CH, and an increase in its transcript level at the same time point. The same tendency was observed in ‘Kordia’ but at a different temporality, where a decrease in methylations were observed at 1,295 CH and the increase in gene expression was observed from 443 CH (**Figure 5**).

Five genes were related to the metabolism of lipids and for example a GDSL esterase/lipase increased its methylation levels from a DMR at 173 CH (‘Royal Dawn’) and 443 CH (‘Kordia’), concomitant with the decrease in gene expression from 348 CH and 1,295 CH, respectively (**Figure 5**). From the phenylpropanoid metabolism, two genes were identified: a putative *PHENOLIC GLUCOSIDE MALONYLTRANSFERASE 1-like* and *FLAVONOID 3-O-GLUCOSYLTRANSFERASE-like*. The latter showed an increase in methylations from a DMR located in an intron at 173 CH (‘Royal Dawn’) and 443 CH (‘Kordia’), followed by a decrease in expression from 348 CH and 1,295 CH, respectively.

The *SHORT VEGETATIVE PHASE* (*SVP*) gene is involved with the regulation of vernalization in *Arabidopsis thaliana* and is an orthologous of the *DAM* genes. In ‘Royal Down’, a DMR located downstream the gene increased its methylation levels at 348 CH, in addition to a decrease in the transcript levels at the same time point. Meanwhile in ‘Kordia’, this increase in methylations was observed from 443 CH and was associated to a decrease in gene expression from 1,295 CH (**Figure 5**). All these results showed that despite the conserved profile of methylations and transcript levels from the thirty genes, patterns were unique to each variety and related to their CR.

### Correlation Between DEGs and DMRs Across All Chilling Conditions

**Figure 6:**
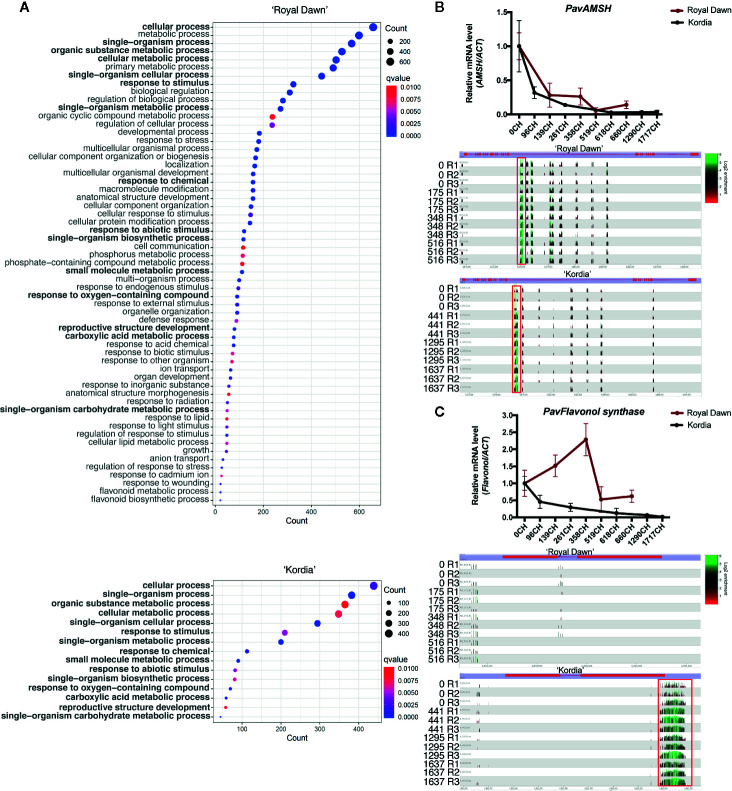
Enrichment of differentially expressed genes that negatively correlated (r <–0.5) with methylation levels during chilling accumulation. **(A)** Gene Ontology terms (p-value <0.01) of biological processes from DEGs associated with DMRs in ‘Royal Dawn’ and ‘Kordia’. The mechanisms in bold represent conserved mechanisms between varieties and the dot size indicates the number of genes as counts. **(B)** Transcript (above) and DNA methylation level (below) of a DMR of approximately 300 bp in an intron of *PavAMSH* (Pav_sc0001014.1_g060.1.mk), gene from the ‘cellular process’ mechanism that was methylated in both varieties. Transcripts are represented as qPCR analysis and gene methylation as enrichment levels from Seqmonk visualizer, where green peaks indicate high levels and black to red indicate low levels of methylation. **(C)** Differential regulation of *PavFLAVONOL SYNTHASE* (Pav_sc0000030.1_g1340.1.mk), gene from the mechanism ‘response to abiotic stimulus’, being methylated in approximately 300 bp of its 3’ end. Transcripts are represented as qPCR (above) and DNA methylation as enrichment (below).

To identify genes that constantly decrease their expression (in RNA-seq and qPCR analysis) together with their closest DMR (up to 2,000 bp upstream and downstream of genes) that may constantly increase its methylation level across all the chilling accumulation conditions, or vice versa, we calculated the Pearson correlation value between RNA and DNA methylation levels through 0 CH, 348 CH and 516 CH for ‘Royal Dawn’ and across 0 CH, 443 CH, 1,295 CH and 1,637 CH for ‘Kordia’. Genes with a Pearson value of r = −0.5 or less were used for further study (**Tables S9** and **S10**). From these genes (r ≤−0.05), we obtained the GO categories and observed an overrepresentation of 60 biological processes in ‘Royal Dawn’, while only 15 processes were in ‘Kordia’ (**Figure 6A**). Afterwards, from the GO analysis we further studied three genes that presented larger DMRs, from 70 bp to 300 bp. From the GO term ‘cellular process’ present in both varieties, we analyzed *PavAMSH-*like (*AMSH-like UBIQUITIN THIOESTERASE 3 ISOFORM X1*) (**Figure 6B**), a gene involved with cellular trafficking. In ‘Royal Dawn’ and ‘Kordia’, the methylation level from the DMR of approximately 300 bp increased at 173 CH and 443 CH respectively. This DMR was located in the intron of *AMSH*-like and correlated with a decrease in the expression of this gene from early stages of chilling accumulation, 139 CH in ‘Royal Dawn’ and 96 CH in ‘Kordia’.

Additionally, from the term ‘response to abiotic stimulus’, which was also present in both varieties, we analyzed a *PavFLAVONOL SYNTHASE-like* gene that showed a variety-specific regulation (**Figure 6C**). In ‘Kordia’, a DMR of approximately 300 bp located at the 3’ end of the gene showed an increase in the methylation levels from 443 CH, coincident with a constant decrease in the expression levels from 96 CH. On the contrary, in ‘Royal Dawn’ this region was not methylated at any time point, and gene expression constantly increased until its downregulation at 519 CH, but still maintaining higher levels than ‘Kordia’. Hence, this variety-specific result is complementary to the conserved profiles of *FLAVONOID 3-O-GLUCOSYLTRANSFERASE-like* and *PHENOLIC GLUCOSIDE MALONYLTRANSFERASE 1-like* mentioned previously for **Figure 5**.

**Figure 7:**
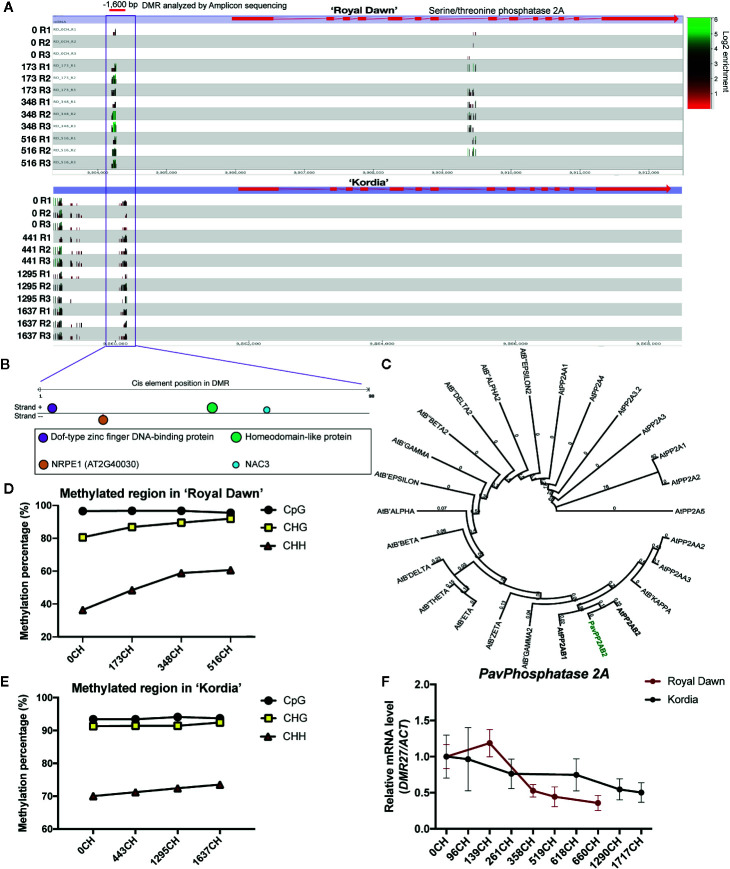
Analysis of a DMR located −1,600 bp from a *PHOSPHATASE 2A* (*PP2AB2*) gene. **(A)** Seqmonk genome browser of the putative *PHOSPHATASE 2A* (Pav_sc0004290.1_g070.1.mk) and its level of DNA methylation including all cytosine context. Methylation levels are observed as log2 enrichment in ‘Royal Dawn’ and ‘Kordia’, where green represents high levels and red, low levels. The red line indicates the DMR analyzed by Amplicon bisulfite sequencing (ABS). **(B)** *In silico* analysis of the DMR sequence in The Plant ChIP-seq Database (PCBase), where color circles indicate the interaction sites with a zinc finger protein (AT1G64620), a homeodomain protein (AT2G40260), NRPE1 (AT2G40030) and NAC3 (AT3G15500). **(C)** Phylogenetic tree considering the aminoacidic sequence of PP2AB2 from sweet cherry (in bold-green) and 25 phosphatases from *Arabidopsis thaliana.* **(D)** Level of DNA methylation as percentage in the DMR analyzed by ABS in ‘Royal Dawn’ and **(E)** ‘Kordia’. **(F)** Transcript levels of *PHOSPHATASE 2A* analyzed by qPCR relative to *Pavβ-ACTIN* using three biological and three technical replicates. Error bars represent standard deviation (SD).

Another gene named *PavPHOSPHATASE 2A* (*PP2A*), which belong to the terms of ‘protein modification process’, ‘biological regulation’ and may be related to the cold-signaling process, also showed a genotype-specific regulation (**Figure 7**). This gene possesses a DMR of 70 bp located ˜1,600 bp upstream that can be targeted and analyzed by ABS (**Figure 7A**). Initially from the MethylC-seq analysis, the methylation level of this DMR increased in ‘Royal Dawn’ at 173 CH, while in ‘Kordia’ this methylation level was maintained across all CH conditions (**Figure 7A**). An *in silico* study of the DMR sequence in The Plant ChIP-seq Database (PCBase) showed the presence of *cis*-regulatory elements for transcription factors Dof1.8 (AT1G64620), homeodomain-like protein (AT2G40260), NAC3 (AT3G15500), and a subunit of the nuclear DNA-dependent RNA polymerase V NRPE1 (AT2G40030) required for RNA-directed DNA methylation (RdDM) (**Figure 7B**). With the aminoacidic sequence of PavPP2A and the related proteins from *Arabidopsis*, we observed an 81% of identity and a closest phylogeny with AtPP2AB2 (AT1G17720), revealing that this gene is highly conserved between these species (**Figure 7C**). Later, by using ABS, this DMR also showed an increase in the methylation levels (from 27.5 to 58.7%) in the CHH context from 173 CH in ‘Royal Dawn’, coincident with the results of MethylC-seq and with the presence of a *cis*-element for NRPE1 (**Figure 7D**). While in ‘Kordia’, methylation in the CHH context was maintained from 70 to 73.5%, suggesting that the regulation of this gene is dependent on the variety (**Figure 7E**). This result also coincided with the transcript profile of *PavPP2A*, which was different for both varieties. In ‘Royal Dawn’, this gene was downregulated from 358 CH and in ‘Kordia’, the transcript level was maintained until 1290 CH, followed by a slight decrease (**Figure 7F**). These changes were also observed from validated RNA-seq data (**Supplementary Figure 5**).

## Discussion

The effect of the environment on epigenetic regulation can induce changes in gene expression that trigger a certain phenotype. The study of environmental epigenetics has been focused in DNA methylation due to its essential role in development, genomic imprinting and silencing of transposable elements (Feil and Fraga, 2012). As a consequence of repeated changes in temperature, epigenetic transitions can arise from individual plants. An example of this is the vernalization process in *Arabidopsis* and dormancy in perennial trees from temperate climates (Chinnusamy and Zhu, 2009; Feil and Fraga, 2012). In this study, we used an epigenomic and transcriptomic approach to elucidate how changes in DNA methylation and gene expression participate in the chilling accumulation process of dormant buds in sweet cherry.

### Chilling Requirement (CR) Variability and Methylome Dynamics in Dormant Floral Buds

Temperate fruit trees can be grown in many regions with different environmental conditions and even within the same region, climate conditions can be different depending on the year, making some traits like CR and flowering date, variables across different locations and seasons (Alburquerque et al., 2008; Castede et al., 2014). During seasons 2015 and 2016, ‘Royal Dawn’ showed a CR that oscillated between 516 CH and 660 CH, respectively (**Figure 1**). On the other hand, ‘Kordia’ trees needed a higher accumulation of chilling in both seasons (1,637 and 1,290 CH), showing higher variability due to different locations of sampling and being consistent with the important effect of the environment over this trait.

The presence of epigenetic modifications related to the environmental conditions of dormancy has been previously reported in cherry, peach, apple, pear (*P. pyrifolia*), almond (*P. dulcis*), and chestnut (*C. sativa*) (Santamaría et al., 2009; Leida et al., 2012; Saito et al., 2015; Kumar et al., 2016; Rothkegel et al., 2017; Prudencio et al., 2018). In this work, the relative levels of methylated cytosines in ‘Royal Dawn’ and ‘Kordia’ at season 2015 showed that the most methylated context was CpG, followed by CHG and CHH (**Figure 2**). These patterns coincide with methylomes from model species like *Arabidopsis* and apple (*Malus domestica* borkh.), which have a higher level of CpG methylation (49–55%), continued by CHG (23–39%) and CHH (12–22%), highlighting that each type of methylation is regulated under different pathways (Lister et al., 2008; Daccord et al., 2017). Methylation in the CG context is maintained during DNA replication by DNA METHYLTRANSFERASE 1 (MET1), and methylation in the CHG context is maintained by a reinforcing loop involving CHROMOMETHYLASE 3 (CMT3) and histone marks (H3K9). On the other side, CHH methylation is carried out by DOMAINS REARRANGED METHYLTRANSFERASE 2 (DRM2) and RNA-directed DNA Methylation (RdDM). In this pathway, small non-coding RNAs target homologous DNA sequences for methylation by DRM2 not only on CHH cytosines, but also on CG and CHG (Law and Jacobsen, 2010).

Considering the tree cytosine contexts, we identified 9,600 DMRs in ‘Royal Dawn’ and 8,535 in ‘Kordia’ across all CH accumulation. This difference is mainly due to the genetic background of each variety, having an important effect on epigenetic modifications. In addition, we observed that most of these DMRs refers to hypermethylations between 0 CH and the following condition of CH accumulation (173 CH in ‘Royal Dawn’ and 443 CH in ‘Kordia’), suggesting that an increase in DNA methylation occurred as an early response to cold temperatures and that the moment in which these changes occur may be even earlier than the sampled points. Concomitant with this, the highest variation in methylation for both varieties was observed in the CHH context, indicating that genes regulated by RdDM may play important roles into an abiotic stress response like temperature. In previous works, hypermethylation was reported during dormancy in chestnut, apple and almond, where the authors observed higher levels of methylation in dormant buds followed by its decrease at bud burst (Santamaría et al., 2009; Kumar et al., 2016; Prudencio et al., 2018). Regarding to another abiotic stress like salinity, in *Populus euphratica*, increases in methylation after salt treatments were dependent on the tissue and most methylations (57.4–66.2%) occurred in CHH cytosines, also suggesting the participation of RdDM as an abiotic stress response (Su et al., 2018). Moreover, RdDM can dynamically and reversibly regulate the expression of adjacent genes involved with stress responses (Matzke et al., 2015). Coincident with this, another study of sweet cherry during chilling accumulation showed an increase in DNA methylation and abundance of matching small interference RNAs, which were associated with RdDM in the promoter of a dormancy-associated MADS-box gene (Rothkegel et al., 2017).

### Chilling Accumulation Modulates the Transcriptome of Sweet Cherry, Which Is Preceded by Changes in Methylation Levels

In response to low temperatures, plants can rearrange their transcriptomes and induce a large number of stress-related genes for cold acclimation. A recent work in sweet cherry showed that buds in the stages of organogenesis, paradormancy, endodormancy and ecodormancy, can be defined by the differential expression of genes involved with specific pathways. Moreover, endodormancy was characterized by pathways of cold response genes, ABA and oxidation-reduction processes (Vimont et al., 2019). In this work, we analyzed changes of the transcriptome during the chilling accumulation stage of endodormancy followed by co-expression analysis (**Figure 3**). According to the PCA, in ‘Royal Dawn’, the overall gene expression was reprogrammed rapidly from 348 CH in response to low temperatures. On the opposite, ‘Kordia’ showed that changes in gene expression started from 443 CH, increased at 1,295 CH and were later maintained until a complete CR at 1,637 CH. Coincident with this, the expression profile from the top 1,000 genes and co-expression analysis, also showed changes from 348 CH in ‘Royal Dawn’ and 1,295 CH in ‘Kordia’. These results suggest a reprogramming that depends on the genetic background of each variety, as reported previously from QTL analysis for the trait of CR (Castede et al., 2014).

In accordance with methylation changes, which occurred from 173 CH in ‘Royal Dawn’ and 443 CH in ‘Kordia’, the reprogramming of the transcriptome was preceded by changes at the methylation level. Regarding this, in ‘Kordia’, 1,219 differentially expressed genes (DEGs) were observed at 443 CH, increasing to 4,291 genes at 1,295 CH and 4,387 genes at 1,637 CH (**Figure 4**). In this sense, the dynamics of DNA methylation as a transcriptional regulator has been widely studied in plants and animals. DNA methylation can regulate gene transcription by directly interfering with the binding of transcription factors to their recognition sequences (Attwood et al., 2002). However, despite this, no direct correlation can be found between MethylC-seq and RNA-seq data because usually, a specific pattern reflects a dynamic regulation of establishment and maintenance and thus, it is necessary to identify which gene was regulated due to a methylation change and which gene was previously regulated by another factor, but being maintained by a methylation change (Zhang et al., 2018).

In this work, an increase from 162 to 186 downregulated genes was associated to hypermethylated regions in ‘Royal Dawn’. In ‘Kordia’, 25 hypermethylated regions overlapped with downregulated genes at 443 CH, increasing to 143 genes at 1,295 CH and 1,637 CH (**Figure 4**). This result showed that most of the DMRs were not associated with genes that significantly change their expression, however, they indicate a connection between DNA methylation, transcript levels and the chilling accumulation process. Hence, patterns of DNA methylation and gene expression modulated by chilling accumulation could help to establish and/or maintain the endodormancy state in sweet cherry.

### Overlapping Between DMRs and DEGs Reveal Conserved Genes Associated to Cold-Sensing and Signaling, Oxidation-Reduction Process, Flowering Regulation, Phenylpropanoid and Lipid Metabolism

From the overlapping between hyper/hypomethylated regions and down/upregulated genes, we searched for those with conserved patterns between ‘Royal Dawn’ and ‘Kordia’ (**Figure 4**). Most of these genes were associated to a cold response, metabolism of lipids, genes of the oxidative-reduction process, metabolism of phenylpropanoids and flowering regulation (**Figure 5**).

Plants cultivated in temperate regions, like sweet cherry, can go through a process of cold acclimation to increase their tolerance to non-lethal low temperatures, which involves a series of physiological, biochemical and molecular changes. One hypothesis associated to the sensing of cold, is the reduction in the fluidity of the cell membrane when exposed to low temperatures, being the first barrier for the environment (Ding et al., 2019). Changes in the fluidity of the membrane are directly correlated with the proportion of desaturated fatty acids, affecting the metabolism of lipids (Martiniere et al., 2011). Another level of cold-sensing is the influx of Ca^2+^, an important messenger of environmental cues. An increase in the Ca^2+^ influx is usually observed seconds after cold treatment and is directly correlated with the upregulation of cold regulated genes, showing that ion channels and electrophysiological responses also mediate the cold sensing (Knight et al., 1996). As an early response to cold, the downregulation of Ca^2+^ dependent proteins in our work was associated to the dissipation of these transcripts with chilling accumulation from 348 CH in ‘Royal Dawn’ and 443–1,295 CH in ‘Kordia’.

In addition, post-translational modifications carried out by kinases and phosphatases respond to Ca^2+^ influx and membrane fluidity in an early response to cold (Sangwan et al., 2001; Teige et al., 2004), explaining the presence of receptor kinases in our results, which also decrease their expression in later CH, associated to increased methylation levels from DMRs. Additional genes identified with similar patterns were *ERF13* and a *HEAT-SHOCK FACTOR*, both downregulated by cold.

The production of reactive oxygen species (ROS) during dormancy has been of increasing interest, since oxidative and respiratory stresses are associated with bud break, suggesting that ROS molecules like H_2_O_2_ may also act as signaling molecules for dormancy (Beauvieux et al., 2018). Additionally, chilling alters protein stabilization, reducing the activity of ROS scavenging enzymes and increasing the oxidative stress (Orvar et al., 2000). In agreement with this, an increased expression of the dehydrogenase *FOLD1* was related to a decrease in methylation, while the opposite pattern was observed in other dehydrogenases, which may be related to the oxidative stress during dormancy.

From the phenylpropanoid metabolism, two genes were identified: a putative *PHENOLIC GLUCOSIDE MALONYLTRANSFERASE 1-like* and *FLAVONOID 3-O-GLUCOSYLTRANSFERASE-like*. Flavonoids are a specific type of phenylpropanoid and are associated with the transport and biosynthesis of auxins, which is an important phytohormone for dormancy regulation. In this sense, it was proposed that the flux of phenylpropanoids in response to environmental cues may be important for growth cessation in winter and growth resumption in spring (Conrad et al., 2019).

Regarding flowering regulation, *SVP* is a repressor of flowering described in *Arabidopsis* and is negatively regulated by low temperatures to allow flowering in spring (Kurokura et al., 2013). In this work, it was observed that *SVP-like* decrease its expression with chilling accumulation, together with an increase in the methylation levels, indicating a similar regulation to *Arabidopsis*.

### Correlation Between DNA Methylation and Transcript Levels of Genes Involved With Cellular Trafficking, Flavonoid Metabolism, and Protein Phosphorylation During Chilling Accumulation

DNA methylation and modifications in the chromatin are important epigenetic marks that help to regulate gene expression, transposon silencing, chromosome interactions and inheritance of traits (Zhang et al., 2018). From genes that showed a negative correlation between their methylation state and transcript levels, we obtained their GO categories and searched for DMRs of greater size (>50 bp) that could be further studied (**Figure 6**). The most represented GO term in both varieties was ‘cellular process’, from which we analyzed a putative *PavAMSH-*like. This gene was downregulated from early CH accumulation and presented an hypermethylation located in an intron, that could be regulating processes like splicing and polyadenylation (Zhang et al., 2018). AMSH is a major deubiquitinating enzyme that hydrolyzes K48- and K63-linked ubiquitin chains, and is essential for vacuole biogenesis, vacuolar trafficking from the Golgi and endocytosis (Isono et al., 2010). Therefore, when exposed to low temperatures, the cellular transport and metabolic activity are decreased, which is coincident with the downregulation of *PavAMSH* (Takahashi et al., 2013).

A second gene, from the GO term ‘response to abiotic stimulus’, named *PavFLAVONOL SYNTHASE-like* showed a genotype-specific regulation, being highly expressed in ‘Royal Dawn’ and downregulated in ‘Kordia’, possibly by an hypermethylation at the 3’ end of the gene present only in ‘Kordia’, indicating that flavonoids may have an epigenetic regulation (**Figure 6C**).

From the ‘protein modification process’ category, *PavPHOSPHATASE 2A* had an hypermethylated region in the CHH context located upstream, correlating (r = −0,98) with a decrease in its expression level in ‘Royal Dawn’(**Figure 7**). The increase in the methylation levels at the CHH context was related to the presence of a putative *cis* element that interacts with NRPE1, the largest subunit of the plant-specific RNA Polymerase V that participates in the RdDM pathway (Greenberg et al., 2010), suggesting that *PavPP2A* may be regulated by this pathway during dormancy in a variety dependent manner. As mentioned previously, in response to low temperatures, protein phosphorylation and the suppression of protein phosphatases, are associated with cold sensing and signaling. For example, in *Arabidopsis*, plants that overexpressed MKK2 (MAP kinase kinase2), presented the up-regulation of proteins from the cold-sensing pathway CBF/DREB1 (C-REPEAT BINDING FACTOR/DEHYDRATION-RESPONSIVE ELEMENT BINDING FACTOR 1), increasing cold tolerance (Teige et al., 2004). Another example is the phosphorylation of ICE1 (INDUCER OF CBF EXPRESSION 1), considered as the master regulator of CBF, activating their expression in response to low temperatures (Ding et al., 2020).

As conclusion, this study provides the first methylome information in sweet cherry during the endormancy process. Changes in the levels of DNA methylation, mostly represented as hypermethylations from early CH, suggested a role for epigenetic modifications in response to low temperatures to increase cold tolerance during endodormancy. In particular, a higher number of DMRs in the CHH context could indicate the participation of RdDM during abiotic stress as a molecular integrator of the environmental cues. In response to low temperatures, the reprograming of the transcriptome was preceded by changes in methylation levels and the integrated data revealed an increase of DEGs that overlap with DMRs during CH accumulation in a temporality that was dependent on the variety. This data established a connection between DNA methylation, transcripts and chilling accumulation during endodormancy. An important role for cold sensing and signaling pathways, lipid and phenylpropanoid metabolism, and oxidative stress was suggested for endodormancy regulation in sweet cherry.

## Data Availability Statement

The datasets presented in this study can be found in online repositories. Raw data is available at NCBI sequence read archive PRJNA610988, PRJNA610989, PRJNA611731 and PRJNA611733 and accession number(s) can be found in the article/**Supplementary Material**.

## Author Contributions

CM, AA, and KR designed the research. KR, ES, and VL-C conducted field work and sampling. JC-M contributed with bioinformatic analysis of MethylC-seq. KR and PS performed experiments and analyzed data of MethylC-seq and Sanger sequencing. LU, ES, PS, and KR conducted amplicon bisulfite sequencing. KR, ES, and LU contributed with RNA-seq experiments, while AR performed bioinformatic analysis of RNA-seq. LU, ES, and KR validated RNA-seq by qPCR. CM conducted statistical analysis. KR wrote the original manuscript. VL-C, CM, and AA edited and reviewed the original manuscript. All authors contributed to the article and approved the submitted version.

## Funding

This study was funded by Corporación de Fomento de la Producción (CORFO 13CTI21520-SP05), Fondo Nacional de Desarrollo Científico y Tecnológico (FONDECYT 1160584) and by a grad student national grant (ANID 21170365).

## Conflict of Interest

The authors declare that the research was conducted in the absence of any commercial or financial relationships that could be construed as a potential conflict of interest.
