# Supplementary material for: Dormant but Active: Chilling Accumulation Modulates the Epigenome and Transcriptome of Prunus avium During Bud Dormancy
Source: Front Plant Sci. 2020 Jul 17;11:1115. doi: 10.3389/fpls.2020.01115 (PMC7380246; doi:10.3389/fpls.2020.01115)
Supplement: Supplementary file 1 [file DataSheet_1.docx]

Supplementary Material

**
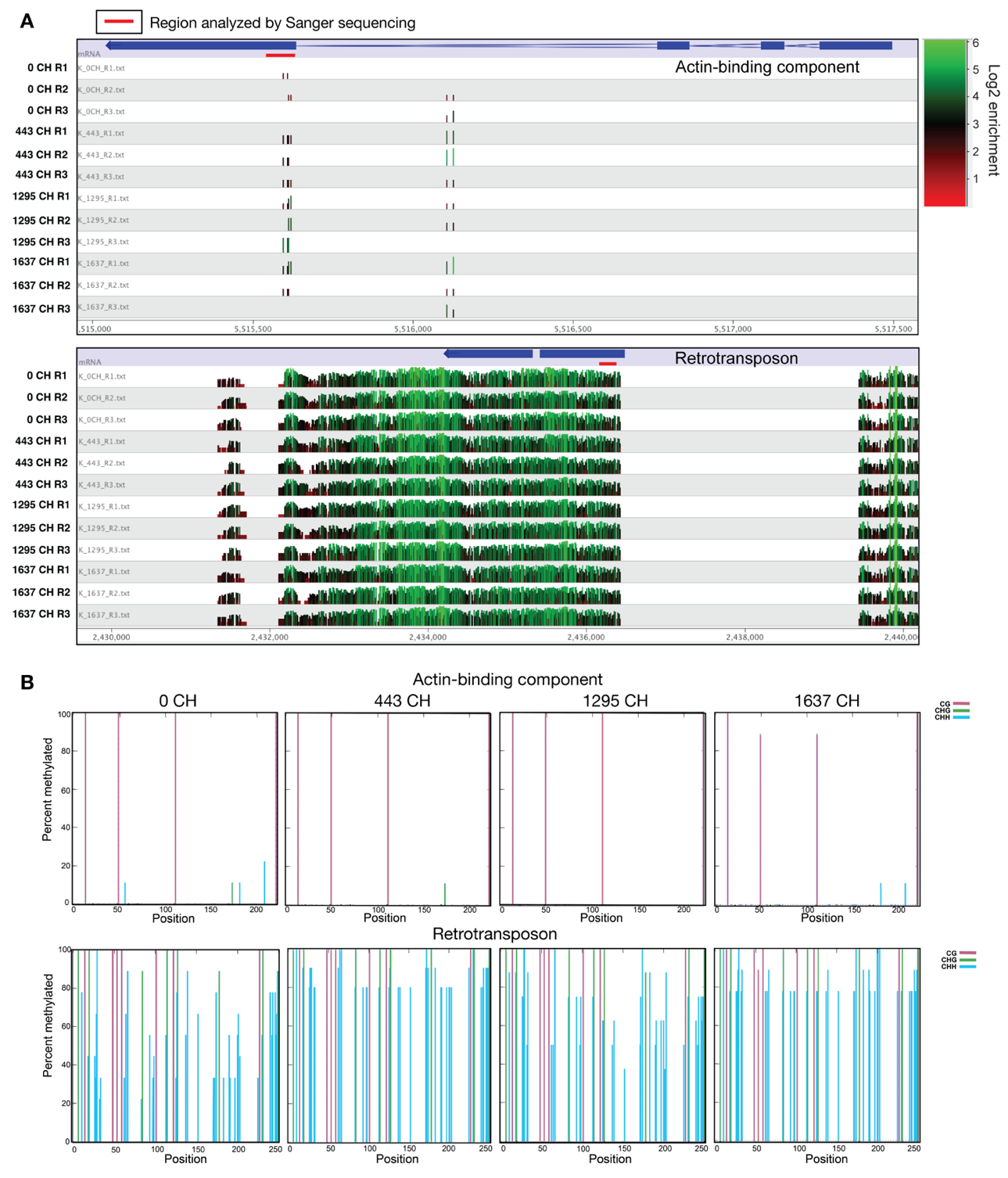
Supplementary Figure 1** Methylation levels of candidate genes from MethylC-seq as controls for methylation in dormant flower buds of *P. avium* ‘Kordia’. **(A)** Seqmonk visualization of methylation in all cytosine context for a putative *ACTIN-BINDING COMPONENT* that was expressed with CH accumulation (above; Pav_sc0002118.1_g070.1.mk) and a putative retrotransposon that was silenced in all the conditions (below; Pav_ sc0000224.1_g040.1.br). Methylation levels are observed as log2 enrichment in ‘Kordia’ for each chilling condition and biological replicate (R1, R2 and R3). Green color represents high levels of methylation and red show low levels. The red line indicates the region analyzed by Sanger bisulfite sequencing. **(B)** Percentage of methylation for each cytosine (*Y*-axis) and tree different contexts (CG, CHG and CHH; H = C, T or A), in a region of *ACTIN-BINDING COMPONENT* (above) and a retrotransposon (below) analyzed by Sanger bisulfite sequencing of ten clones per condition.

**
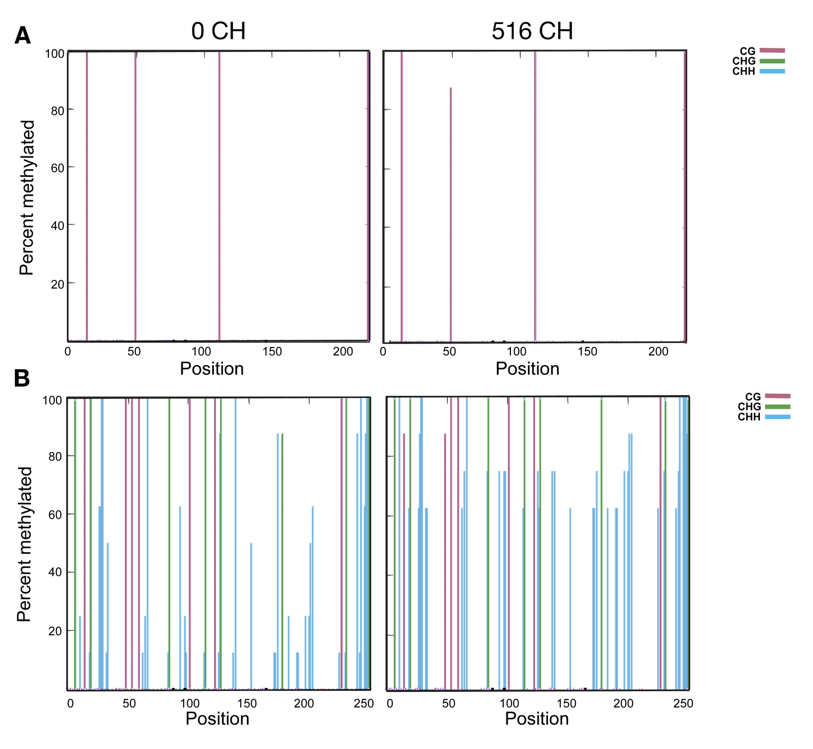
**

**Supplementary Figure 2** Methylation levels of candidate genes from the methylome as controls for methylation in dormant flower buds of *P.avium* ‘Royal Dawn’. Percentage of methylation of each cytosine (*Y*-axis) for tree different contexts (CG, CHG and CHH; H = C, T or A) in a region analyzed by Sanger bisulfite sequencing of 10 clones. **(A)** Putative *ACTIN-BINDING COMPONENT* (Pav_sc0002118.1_g070.1.mk) **(B)** Retrotransposon (Pav_ sc0000224.1_g040.1.br).


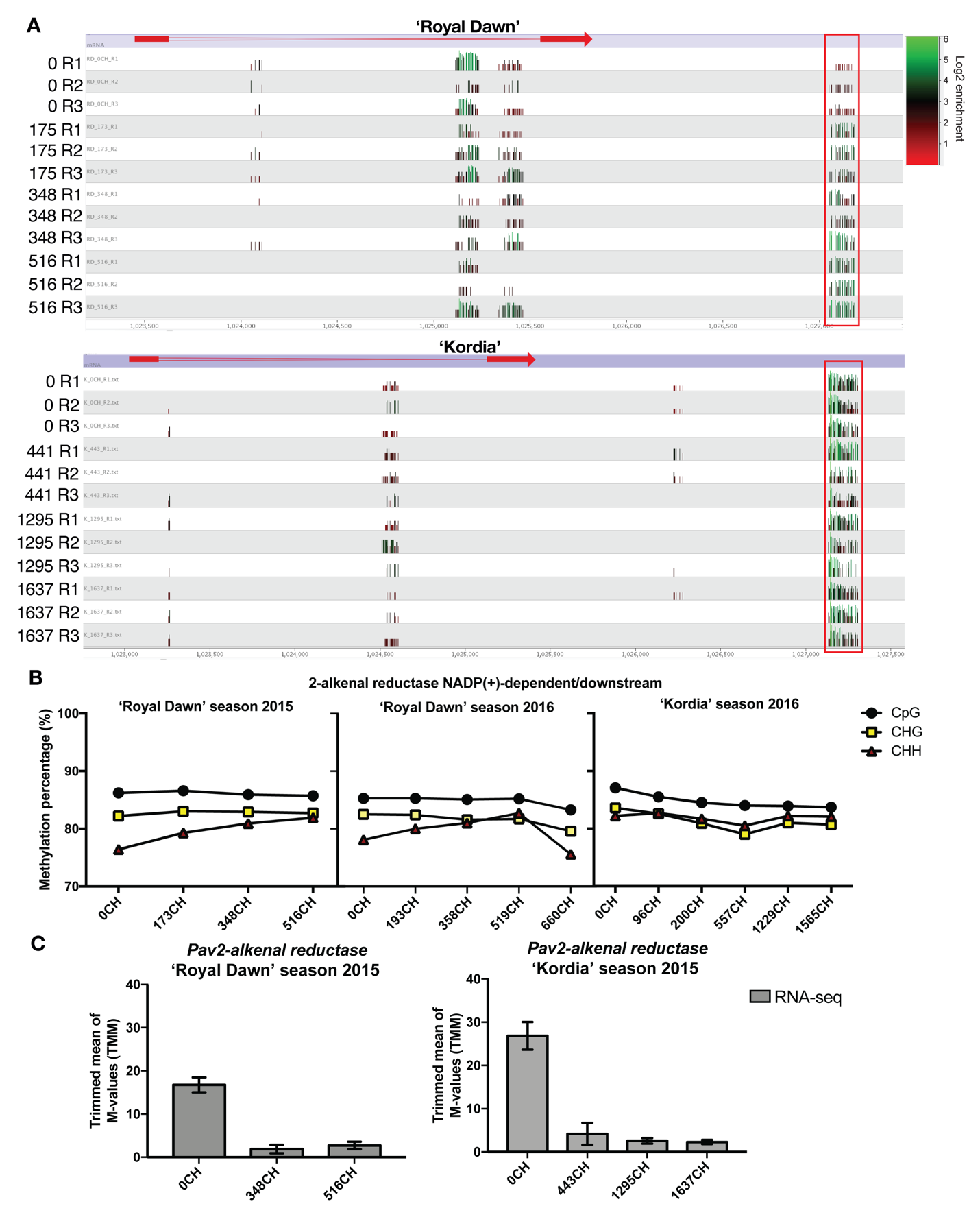


**Supplementary Figure 3** Methylation levels from a candidate DMR close to a *NADPH-DEPENDENT OXIDOREDUCTASE 2-ALKENAL* gene by Amplicon Bisulfite Sequencing (ABS). **(A)** Enrichment of DNA methylation in all cytosine contexts by MethylC-seq in ‘Royal Dawn’ and ‘Kordia. Green color represents high levels of methylation and red show low levels. The red square indicates a region of 180 bp analyzed by ABS. **(B)** Percentage of DNA methylation obtained from ABS of the DMR in ‘Royal Dawn’ for two consecutive seasons and ‘Kordia’. **(C)** Transcript level of *2-ALKENAL REDUCTASE NADPH-DEPENDENT* gene (Pav_ sc0001472.1_g110.1.mk) from RNA-seq data normalized as trimmed mean of M-values (TMM).

**
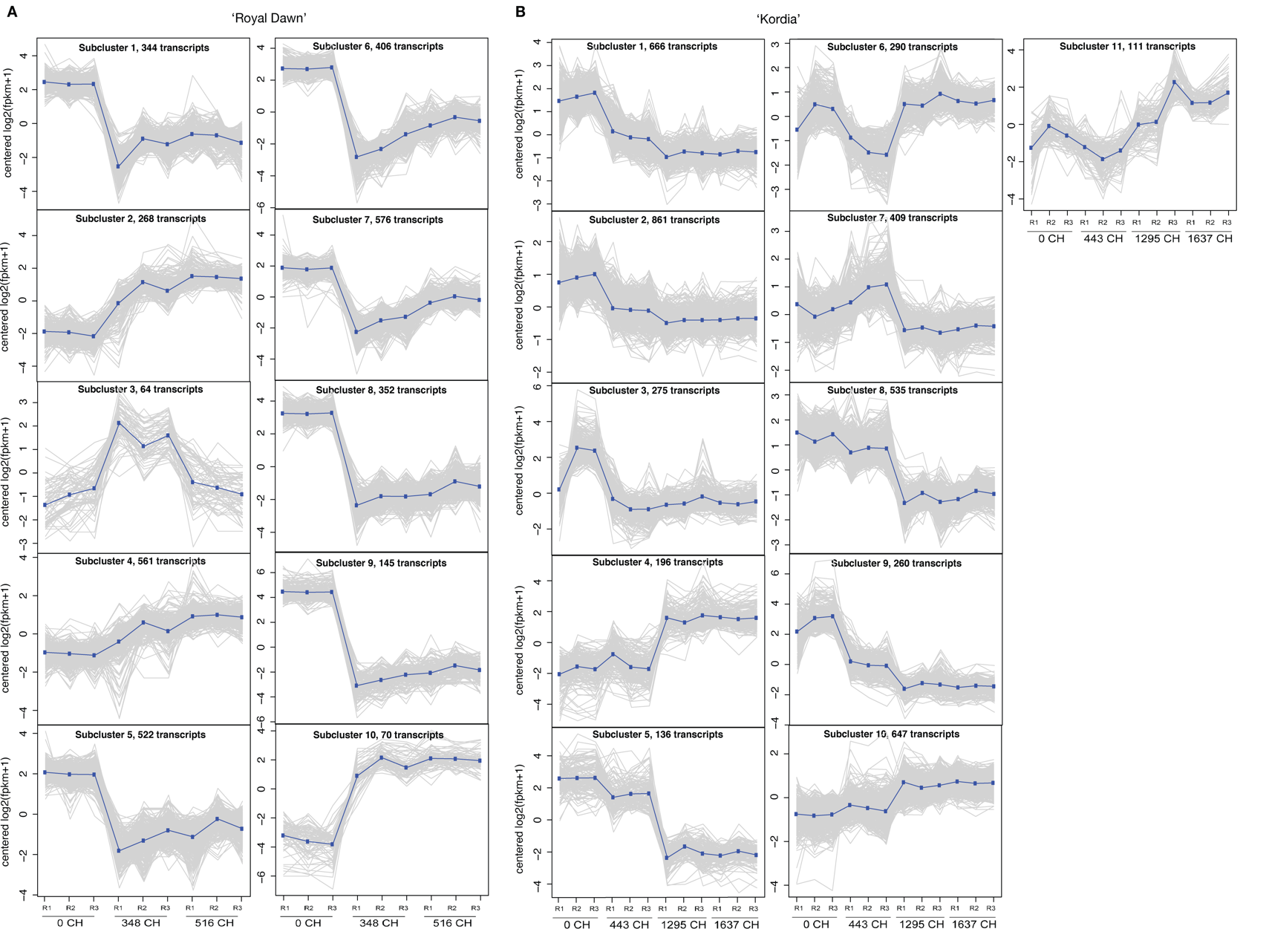
**

**Supplementary Figure 4** Profile of subclusters representing the average expression from differentially expressed genes (DEGs). **(A)** Expression profile of overall genes in ‘Royal Dawn’. **(B)** Overall gene expression profile in ‘Kordia’.

**
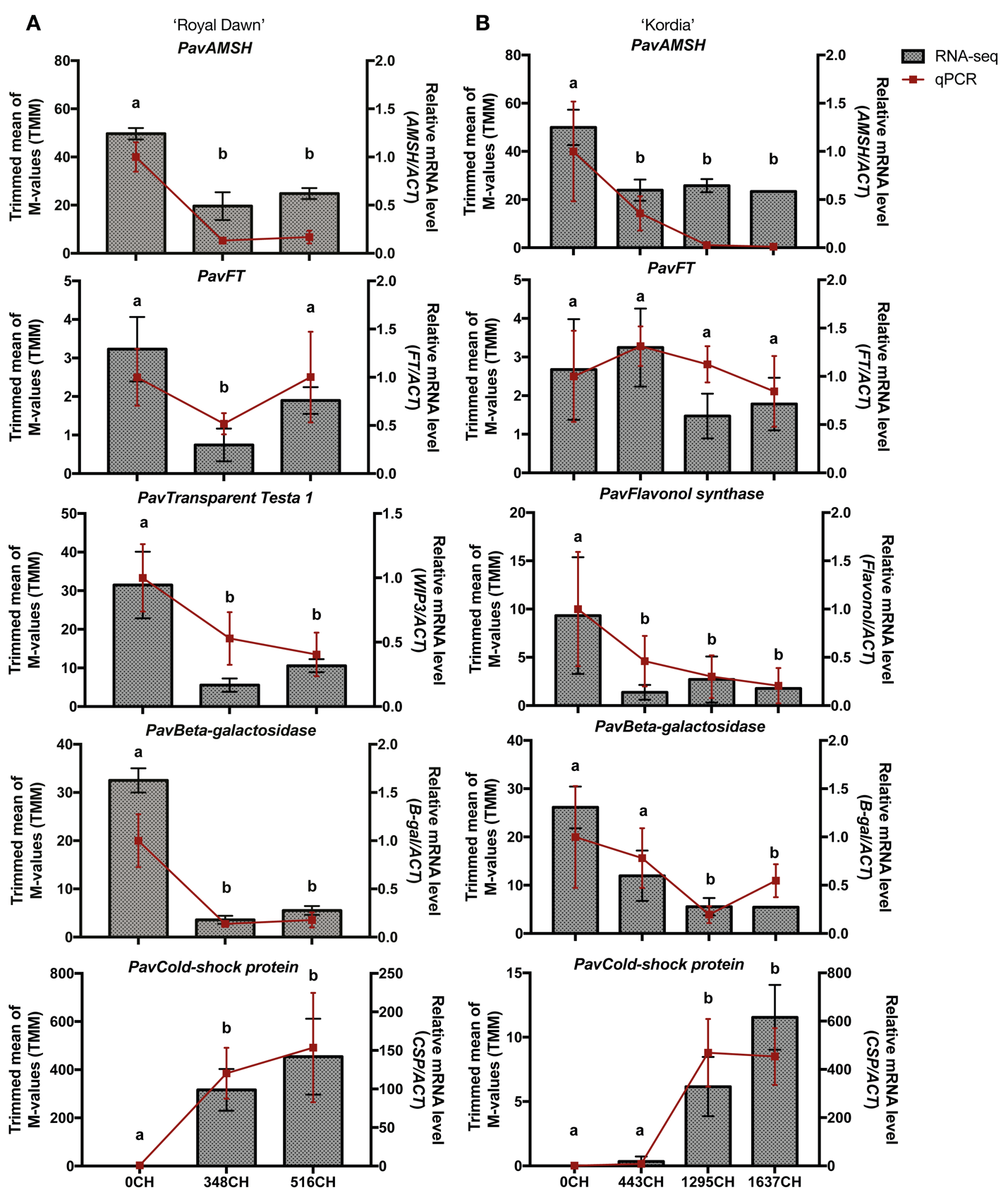
**

**Supplementary Figure 5** Expression levels by RNA-seq and qPCR during chilling accumulation in sweet cherry floral buds. **(A)** Expression levels of *AMSH-like* (Pav_sc0001014.1_g060.1.mk), *FLOWERING LOCUS T* (Pav_sc0001774.1_g100.1.mk), *PavTRANSPARENT TESTA* *1* (Pav_sc0000977.1_g060.1.mk), *FLAVONOL SYNTHASE-like* (Pav_sc0000030.1_g1340.1.mk), *β-GALACTOSIDASE* (Pav_sc0000479.1_g010.1.mk) and *COLD-SHOCK PROTEIN-like* (Pav_sc0001862.1_g360.1.mk). Transcript levels were analyzed by RNA-seq (bars represented as TMM), and real-time qPCR (red line) relative to *Pavβ-ACTIN* using three biological and three technical replicates for ‘Royal Dawn’ and **(B)** ‘Kordia’. Letters represent significant differences (p-value ≤ 0.05) between conditions using a one-way ANOVA test. Error bars represent standard deviation (SD) between 3 biological and 3 technical replicates.
